# Free Radicals Scavenging Capacity, Antidiabetic and Antihypertensive Activities of Flavonoid-Rich Fractions from Leaves of *Trichilia emetica* and *Opilia amentacea* in an Animal Model of Type 2 Diabetes Mellitus

**DOI:** 10.1155/2014/867075

**Published:** 2014-01-28

**Authors:** Kiessoun Konaté, Kassi Yomalan, Oksana Sytar, Patrice Zerbo, Marian Brestic, Van Damme Patrick, Paul Gagniuc, Nicolas Barro

**Affiliations:** ^1^Unit of Formation in Sciences Applied and Technological (UFR/SAT) and Institute of Sciences of the Environment and the Rural Development (ISEDR), Polytechnic University of Dédougou, Burkina Faso; ^2^Laboratory of Biochemistry and Applied Chemistry, University of Ouagadougou, 09 P.O. Box 848, Ouagadougou 09, Burkina Faso; ^3^Laboratory of Animal Physiology, UFR Bioscience, University of Felix Houphouet Boigny of Abidjan, 22 P.O. Box 582, Abidjan 22, Cote D'Ivoire; ^4^Department of Plant Physiology and Ecology, Taras Shevchenko National University of Kyiv, Volodymyrs'ka Street 64, Kyiv 01601, Ukraine; ^5^Laboratory of Plant Ecology and Biology, University of Ouagadougou, 09 P.O. Box 848, Ouagadougou 09, Burkina Faso; ^6^Department of Plant Physiology, Slovak Agriculture University in Nitra, A. Hlinku 2, 94976 Nitra, Slovakia; ^7^Laboratory of Tropical and Subtropical Agronomy and Ethnobotany, Department of Plants Production, Faculty of Biosciences Ingineering, Gent University, Belgium; ^8^Institute of Genetics, University of Bucharest, 060101 Bucharest, Romania; ^9^Laboratory of Biochemistry and Molecular Genetics Microbial, University of Ouagadougou, 03 P.O. Box 7131, Ouagadougou 03, Burkina Faso

## Abstract

*Trichilia emetica* and *Opilia amentacea* traditional Burkinabe medicinal plants were investigated to determine their therapeutic potential to inhibit key enzymes in carbohydrate metabolism, which has relevance to the management of type 2 diabetes. *In vitro* and *in vivo* antioxidant and antihypertensive potential and antilipidemia and antihyperglycemia activities in an animal model of type 2 diabetes mellitus have been studied. The antioxidant activity of the flavonoids from leaves of *Trichilia emetica* and *Opilia amentacea* has been evaluated using **β**-carotene-linoleic acid system, 1,1-diphenyl-2-picrylhydrazyl inhibitory activity, chelation of iron (II) ions, and lipid peroxidation which showed more pronounced antioxidant capacities of *Trichilia emetica*. Total cholesterol concentrations decreased in an animal model of type 2 diabetes mellitus under effects of flavonoid-rich fractions from leaves of *Trichilia emetica* and *Opilia amentacea* has been observed. Extract of flavonoid-rich fractions from *Trichilia emetica* shown maximum radical scavenging activity and possessed marked antiamylase activity which may be due to the presence of certain secondary metabolites. Suggested better antihyperglycemia, antilipidemia, and antihypertensive properties of flavonoid-rich fractions from *Trichilia emetica* compared to the extract of *Opilia amentacea* are demonstrating antidiabetic potential of *Trichilia emetica* as therapeutic targets for the management of type 2 diabetes.

## 1. Introduction

Diabetes mellitus is a common metabolic disorder characterized by hyperglycemia, glycosuria, polyurea, and polydipsia induced by insulin deficiency [1] and insulin resistance [2]. Diabetes mellitus is an important metabolic syndrome. The increasing worldwide incidence of diabetes mellitus in adults constitutes a global public health burden. At least, diabetes mellitus is possibly the world's fastest growing metabolic disorder, and as the knowledge of the heterogeneity of this disorder increases, so does the need for more appropriate therapy [3]. The World Health Organization (WHO) estimates that currently more than 180 million people worldwide have diabetes and it is likely to double by 2030, with India, China, and United States predicted to have the largest number of affected individuals [4, 5]. Diabetes mellitus is treated by using oral hypoglycemic agents such as sulphonylureas, biguanides, meglitinides, and *α*-glucosidase inhibitors. Hyperglycemia, a condition characterized by an abnormal excess of sugar in the blood, has been linked to the onset of type 2 diabetes mellitus and associated cardiovascular complications including hypertension [6, 7]. In effect, stress-related disease such as hypertension or high blood pressure has been considered as a high risk factor cardiovascular disease. Twenty percent of the world's population suffers from hypertension [8]. Experimental evidence suggests that antihypertensive or angiotensin I-converting enzyme (ACE) inhibitor treatment can offer a clinical advantage in hypertension [9]. ACE plays a key physiological role in the control of blood pressure by virtue of the rennin-angiotensin system [10]. Captopril (d-3-mercapto-2-methylpranory-l-proline), enalapril, and lisinopril have been developed and used as clinical antihypertensive drugs. Although synthetic ACE inhibitors, including captopril, are remarkably effective as antihypertensive drugs, they cause adverse side effects, such as coughing, allergic reactions, taste disturbances, and skin rashes. Therefore, research and development to find safer, innovative, and economical ACE inhibitors is necessary for the control of blood pressure.

Although a few synthetic antidiabetic drugs are available to combat the impaired insulin secretion, insulin resistance, and hyperglycemia that characterize type 2 diabetes mellitus, some of these drugs can have negative side effects at high doses [11, 12]. A major focus of current antidiabetic research is the development of antihyperglycemic agents that are safe and free of negative side effects. Many plants and their active chemical compounds have demonstrated activity in the treatment of various disorders [13]. According to ethnobotanical information, more than 800 plants are used as traditional remedies in one or other form for the treatment of diabetes [14]. The management of diabetes without any side effects is still a challenge; therefore, plants continue to play an important role in the discovery of new compounds for the treatment of this disease. The management of diabetes can be achieved by reducing postprandial hyperglycemia by delaying the activities of the enzymes *α*-amylase and *α*-glucosidase which are responsible for the digestion of carbohydrates and absorption of glucose in the digestive tract, respectively [15, 16]. Drugs derived from natural products have played a major role in the development of pharmaceutical treatments for diabetes. Metformin, the single most prescribed agent for the treatment of diabetes, originated from herbal medicine [17, 18]. A plant-derived antidiabetic agent galegine was isolated from *Galega officinalis*. Experimental and clinical evaluations provided the pharmacological and chemical basis for the subsequent discovery of metformin [17, 19]. 1-Deoxynojirimycin (DNJ), a potent *α*-glucosidase inhibitor, was isolated from the water extract of leaves of the mulberry tree (*Morus alba* L.) [20]. There are many cellular biochemical pathways and environmental toxins which produce reactive oxygen species (ROS) [21] and contribute to the development of diseases such as cancer, cardiovascular disorders, diabetes, cataracts, and many neurodegenerative diseases [22]. Many studies have confirmed that plants and foods rich in polyphenolic content are effective scavengers of free radicals, thus helping in the prevention of these diseases through their antioxidant activity [23]. Antioxidants which are present in plants, herbs, and dietary sources help in preventing vascular diseases in diabetic patients [24]. Tannins and flavonoids are the secondary metabolites in plants considered to be the natural source of antioxidants which prevent destruction of *β* cells and diabetes-induced ROS formation [25]. Thus, it is a good strategy to manage diabetes as a whole with plants which show good enzyme inhibitory and antioxidant activities [26].

*Trichilia emetica* and *Opilia amentacea* are native to sub-Saharan Africa and are essentially tropical in origin. In the western part of Burkina Faso, the leaves of these plants are used to treat cardiovascular diseases. They possess hypotensive, hypolipidemic-delite, antioxidant, antibacterial and anti-inflammatory properties [27]. But none have reported on their antioxidant, antidiabetic, and antihypertensive properties of flavonoid-rich fractions. Lack of scientific data to support these claims prompted this study which was therefore aimed at assessing the possible antioxidant, antidiabetic, and antihypertensive properties of flavonoid-rich fractions from the leaves of these plants in models using rats in order to provide a scientific basis for the traditional use of this plant for better management of type 2 diabetes.

## 2. Material and Methods

### 2.1. Plant Materials

Fresh leaves of *Trichilia emetica* and *Opilia amentacea* were collected in October 2011 in Ouagadougou, capital of Burkina Faso, with gardeners. The plants were identified in the Laboratory of Biology and Ecology, University of Ouagadougou, where a voucher specimen was deposited.

### 2.2. Animals Handling

Swiss NMRI mice (25–30 g) and adult albinos Wistar rats (160–200 g) of both sexes were used for this study. All animals were housed in cages under controlled conditions of 12 h light/and 12 h without light and 25°C. They received pellets of food enriched with 20% protein and water ad libitum. They were deprived of food for 15 h (but with access to drinking water) and weighed before the experiments. Experiments on the animals were performed according to the protocols already approved by the Institute of Health Sciences Research/University of Ouagadougou (Burkina Faso) and met the international standards for animal study [28].

### 2.3. Chemicals

Streptozotocin and alloxan monohydrate were purchased from Sigma (Germany) and all other chemicals and reagents used in this study were of analytical grade and were purchased from Sigma Chemical Co. (St. Louis, MO). Glibenclamide, rabbit lung dehydrated by acetone, and captopril were purchased from Sigma-Aldrich, USA.

### 2.4. Preparation of Extracts for Acute Toxicity Study

100 grams of leaves (powdered plant materials) dried in laboratory condition were extracted with 500 mL of acetone 80% (400 mL acetone + 100 mL water) for 24 h under mechanic agitation (SM 25 shaker, Edmund BÜHLER, Germany) at room temperature. After filtration, acetone was removed under reduced pressure in a rotary evaporator (BÜCHI, Rotavopor R-200, Switzerland) at approximately 40°C and freeze-dried (Telstar Cryodos 50 freeze-dryer). The extract was weighed before packing in waterproof plastic flasks and stored at 4°C until use.

### 2.5. Flavonoids Extraction

The fresh harvested plant materials (100 grams of leaves) were dried in the laboratory at room temperature (20–25°C); afterwards samples were ground to pass a sieve of 0.3 mm. Flavonoids were extracted with aqueous acetone (80%, v/v). The extracts were then washed with hexane to remove chlorophyll and other low molecular weight compounds. Acetone was evaporated and then the aqueous extracts with ethyl acetate is used to separate by sequential liquid-liquid extraction. The flavonoid-rich fractions were lyophilized and stored at 22°C prior to biological tests. For the tests, lyophilized sample was dissolved with 10% DMSO in water at the desired concentration.

### 2.6. Antioxidant Capacity of Flavonoids

#### 2.6.1. *β*-Carotene-Linoleic Acid Assay

The antioxidant activity of the flavonoids was evaluated using *β*-carotene-linoleic acid system according to [29]. In short, 1 mL of *β*-carotene solution in chloroform (0.2 mg/mL) was pipetted into a round-bottom flask. To the solution, 20 mg of linoleic acid and 200 mg of Tween 40 were added. After removing chloroform in a rotary evaporator, 50 mL of aerated distilled water was added to the oily residue. Aliquots (5 mL) of thus obtained emulsion were transferred to a series of tubes containing 2 mg of extract or 0.5 mg of butylated hydroxyanisole (BHA) (positive control). Emulsion without antioxidant served as control. After addition of the emulsion to the tubes, they were placed in a water bath at 50°C for 2 h. During that period, the absorbance of each sample was measured at 470 nm at 15 min intervals, starting immediately after sample preparation (*t* = 0 min) until the end of the experiment (*t* = 120 min). The rate of *β*-carotene bleaching (*R*) for the extracts, BHA and water, was calculated according to first-order kinetics. The percent of antioxidant activity (ANT) was calculated as described in [30], using the equation

(1) $$\begin{array}{c} \text{ANT}=\frac{\left(R_{\text{Control}}-R_{\text{Sample}}\right)}{R_{\text{Control}}}\times 100, \end{array}$$

where *R*_Control_ and *R*_Sample_ are average bleaching rates of water control and antioxidant (flavonoids or BHA), respectively.

#### 2.6.2. DPPH Radical-Scavenging Activity

The scavenging effect for DPPH free radical was monitored as described in [31] with minor modification. Briefly, 1.0 mL of 0.16 mM DPPH methanolic solution was added to 1.0 mL of either methanolic solution of extract (sample) or methanol (control). The mixtures were vortexed and then left to stand at room temperature in the dark. After 30 min absorbance was read at 517 nm. Radical-scavenging activity (RSA) for DPPH free radical was calculated using the following equation:

(2) $$\begin{array}{c} \text{RSA}=\frac{\left(A_{\text{Control}}-A_{\text{Sample}}\right)}{A_{\text{Control}}}\times 100, \end{array}$$

where *A*_Control_ is the absorbance of the methanol control and *A*_Sample_ is the absorbance of the flavonoids. Synthetic antioxidant, BHA, was used as positive control. DPPH radical-scavenging activity was calculated as the concentration that scavenges 50% of DPPH free radical and thus has RSA = 50% (EC_50_).

#### 2.6.3. Chelating Activity (ChA)

The chelation of iron (II) ions was studied as described by [32]. An aliquot of the extract in methanol (1.3 mL) was added to 100 *μ*L of 2 mM ferrous chloride. After 5 min, the reaction was initiated by adding 200 *μ*L of 5 mM ferrozine. Following 10 min incubation at room temperature, the absorbance at 562 nm was recorded. For preparation of control, 1.3 mL of methanol was used instead of polyphenols solution. EDTA was used as a chelating standard. The Fe^(2+)^-chelating activity chelating activity (ChA) was calculated using the equation below:

(3) $$\begin{array}{c} \text{ANT}=\frac{\left(A_{\text{Control}}-A_{\text{Sample}}\right)}{A_{\text{Control}}}\times 100, \end{array}$$

where *A*_Control_ is the absorbance of the negative control (solution to which no flavonoid was added) and *A*_Sample_ is the absorbance of the extract solution. Chelating activity was expressed as ChEC_50_, the concentration that chelates 50% of Fe^2+^ ions and thus has ChA = 50%.

#### 2.6.4. Inhibition of Lipid Peroxidation

Liver of male Wistar rats (160–180) was excised and homogenized (1% w/v) in 0.154 mol/L KCl solution. The homogenate was centrifuged at 3000 rpm at 4°C for 10 min and supernatant was used for the assay. Peroxidation of the liver homogenate was induced by FeCl_2_-H_2_O_2_ [33]. Briefly, 1% liver homogenate was incubated with 0.5 mmol/L of each of FeCl_2_ and H_2_O_2_ with or without flavonoid-rich fractions (50 *μ*g/mL). After incubation at 37°C for 60 min, the formation of malondialdehyde (MDA) was measured at 535 nm [33]. BHT served as positive control. The equation is

(4) $$\begin{array}{c} \text{Inhibition}\;\text{of}\;\text{peroxidation}\;\left(\%\right)=\left[\frac{\left(\mathrm{AC}-\text{AA}\right)}{\mathrm{AC}}\right]\times 100, \end{array}$$

where AC is the absorbance of the control (without any treatment) and AA is the absorbance of the antioxidants.

### 2.7. *In Vitro* Antihyperglycemia

#### 2.7.1. Amylase Inhibition Screening Assay

The *α*-amylase inhibitory assay was modified from [34]. Twenty *μ*L of porcine pancreatic *α*-amylase solution (EC 3.2.1.1; equivalent to 3000 U in 50 mM phosphate buffer, pH 6.9) was mixed with 15 *μ*L of plant extract and incubated at 37°C for 45 minutes. After incubation, the mixture was applied to a sterile paper disc and placed onto the center of Petri plates containing medium consisting of 1% (w/v) agar and 1% (w/v) starch in distilled water. Plates were allowed to stand for 3 days at 25°C then stained with iodine and allowed to stand for 15 min. The diameter of the clear zone was measured and used to calculate the amylase inhibitory activity. As a control, the enzyme was mixed with the solvent in which the plants were extracted (ethanol) and applied onto the sterile disc. Results were expressed as

(5) Percentage  (%)  amylase  inhibition=  {(diameter  of  control−diameter  of  sample)diameter  of  control}×100.

#### 2.7.2. Amylase Inhibition Assay by Quantitative Starch Hydrolysis

The *α*-amylase inhibitory activity was determined [35] using porcine pancreatic *α*-amylase solution (EC 3.2.1.1) type VI B. To 125 *μ*L of different plant extract concentrations (range 1.56 *μ*g/mL to 500 *μ*g/mL), *α*-amylase solution (0.5 mg/mL in 0.02 M sodium phosphate buffer) was mixed and the reaction mixture was preincubated for 10 minutes at room temperature. The reaction mixture was diluted by adding 5000 *μ*L of distilled water. The generation of maltose was quantified by measuring the absorbance at 540 nm of 3-amino-5-nitrosalicylic acid (from reduction of 3,5-dinitrosalicylic acid) [36] using a UV-visible spectrophotometer. The control was buffer-treated in the same way as plant samples. The standard used was acarbose (concentration range 1.56 *μ*g/mL to 500 *μ*g/mL). Results were expressed as

(6) Percentage  (%)  inhibition ={(absorbance  of  control540 nm−absorbance  of  samples540 nm)absorbance  of  control540 nm}  ×100.

#### 2.7.3. Glucosidase Inhibition Assay

The *α*-glucosidase inhibition assay has been modified from [37] using yeast *α*-glucosidase (EC 2328898). A volume of 25 *μ*L of plant extract (range 0.35 *μ*g/mL to 100 *μ*g/mL) was mixed with 50 *μ*L of *α*-glucosidase enzyme 0.1 U/mL in 0.1 M potassium in 96 well plates and incubated at 37°C for 30 minutes. After preincubation, 25 *μ*L of 5 mM pNPG in 0.1 M phosphate buffer was added to each well and the reaction mixture was incubated again at 37°C for 30 minutes. Thirty *μ*L of 0.1 M sodium carbonate solution was added to the previous reaction mixture and incubated again for 20 minutes at 37°C. Before and after incubation, the absorbance was measured at 405 nm and compared to the control that contained 25 *μ*L of buffer solution instead of polyphenols solution. The standard used was acarbose (concentration range 0.35 *μ*g/mL to 100 *μ*g/mL). The *α*-glucosidase activity was determined by measuring release of p-nitrophenol from p-nitrophenyl *α*-D-glucopyranoside [38]. The *α*-glucosidase inhibitory activity was expressed as

(7) Percentage  (%)  inhibition ={(absorbance  of  control405 nm−absorbance  of  samples405 nm)absorbance  of  control405 nm}  ×100.

### 2.8. *In Vitro* Antihypertensive Profile by Measurement of ACE Inhibitory Activity

#### 2.8.1. Assay Buffer

HEPES (297.5 mg, 50 mmol/L), NaCl (438.75 mg, 300 mmol/L), and Na_2_SO_4_ (1420 mg, 400 mmol/L) were added to a 25 mL volumetric flask (amount and final concentration given in parentheses). After dissolving in 20 mL of distilled water containing 50 mL of saturated NaOH solution, the pH was made up with distilled water. Phosphate buffer (100 mmol/L) was prepared by dissolving 340.2 mg of anhydrous potassium phosphate in 20 mL of distilled water, adjusted to pH 8.5 with 10% NaOH solution, and made up to 25 mL.

#### 2.8.2. Stock and Working Solution of Rabbit Lung Dehydrated by Acetone

The stock solution was prepared as described previously [39] by dissolving 2 g of rabbit lung dehydrated powder in 10 mL of 50 mmol/L phosphate buffer (pH 8.3). The stock solution was highly active and stable for at least 3 months under refrigeration (2–6°C). Working solution (1 g/10 mL) was freshly prepared by diluting the stock solution in the phosphate buffer before performing the assays.

#### 2.8.3. Substrate Solution

200 mg of hippuryl-glycyl-glycine was dissolved in 4 mL of 1 mol/L ammonium hydroxide solution. After complete dissolution, the volume was increased to 6.8 mL with distilled water.

#### 2.8.4. TNBS Solution

TNBS (2030 *μ*L) was added to a 5 mL volumetric flask and the volume was made up with distilled water to obtain a final concentration of 60 mmol/L. The solution was stored at −20°C and used within 3 months.

#### 2.8.5. Preparation of Flavonoid-Rich Fractions

For antihypertensive screening, the flavonoid-rich fractions were dissolved in 20% methanol and 80% HEPES to a concentration of 5 mg/mL.

#### 2.8.6. Colorimetric Methanol for ACE Inhibition Assay [40]

Ten microlitres of rabbit lung solution (1 g/10 mL) were added to an Eppendorf tube containing 10 *μ*L of flavonoid-rich fractions solution (5 mg/mL) to be tested, or 10 *μ*L of 50 mmol/L phosphate buffer (pH 8.3) (negative control), or 10 *μ*L of captopril solution (5 mg/mL) (positive control). The mixture was homogenized and preincubated for 5 min at 37°C. The enzyme reaction was initiated by adding 60 *μ*L of the assay buffer and 30 *μ*L of the substrate solution. After homogenization, the mixture was incubated for 35 min at 37°C. The reaction was stopped by the addition of 100 *μ*L of sulfuric acid (0.33 mmol/L); the Eppendorf tube was shaken 10 s after the addition of 1000 *μ*L of distilled water. In the sequence, the mixture was centrifuged at 2000 rpm for 10 min. An aliquot of the supernatant (75 *μ*L) was placed on a microtitre plate and mixed with 100 *μ*L of phosphate buffer (100 mmol/L, pH 8.5) and 5 *μ*L of TNBS solution. The plate was kept in the dark at room temperature for 20 min. Its absorbance was read in a microtitre plate reader (Spectramax 340 PC tunable microplate reader) at 415 nm against a blank solution prepared similarly but without adding sodium tungstate and sulfuric acid solutions to the mixture. Assays were performed in triplicate. Calculation of ACE inhibition on a percentage basis was done using the following equation:

(8) $$\begin{array}{c} \text{Inhibition}\;\left(\%\right)=\frac{\left[100-\left(\text{AI}\times 100\right)\right]}{\mathrm{AC}}, \end{array}$$

where AI is the measured absorbance at 415 nm in the presence of an inhibitor and AC is the absorbance of the blank solution.

### 2.9. Acute Toxicity Study of Aqueous Acetone Extracts

Swiss mice (male and female) were randomly divided into 7 groups (1 control group and 6 treated groups) of 6 animals (3 males and 3 females). The control group received water containing 10% dimethylsulfoxide (DMSO) administered intraperitoneally. The aqueous acetone extracts of *Trichilia emetica* and *Opilia amentacea *suspended in 10% DMSO were administered intraperitoneally at doses of 1, 2, 2.5, 3, 4, 5, and 6 g/kg [35]. The general behaviour of the mice was observed for 120 min after the treatment. The animals were observed for morbidity and mortality once a day for 14 days. The number of survivors after the 14 days period was noted. The toxicological effect was assessed on the basis of mortality for 14 days, which was expressed by the median lethal dose value (Lethal Dose 50 or LD_50_) estimated from the regression of log-probit mortality rate [41].

### 2.10. *In Vivo* Antihyperglycemia and Hypolipidaemia Potential of Flavonoids

#### 2.10.1. *In Vivo* Antihyperglycemia

*(1) Induction of Diabetes.* Alloxan monohydrate was first weighed individually for each animal according to its weight and then solubilized with 0.2 mL saline just prior to injection. Diabetes was induced by injecting it at a dose of 100 mg/kg body weight intraperitoneally. After 1 hr of alloxan administration, the animals were got food and 5% dextrose solution was also given in feeding bottle for a day to overcome the early hypoglycemic phase. The animals were kept under observation and after 48 hr, blood glucose was measured. One group served as a control which received vehicle alone. The diabetic rats (glucose level >150 mg/dL) were separated and divided into four different groups for experimental study [42].

*(2) Experimental Design*

*(i) Acute Treatment and Subacute Treatment.* Normal rats are kept into group 1; diabetic induced rats are grouped into groups 2, 3, 4, and 5. Each group contains six rats: group I: normal control (saline); group II: diabetic control (saline); group III: standard (glibenclamide 10 mg/kg); group IV: test-dose (100 mg/kg of flavonoid-rich fractions from *Trichilia emetica* and *Opilia amentacea*); group V: test-dose (300 mg/kg of flavonoid-rich fractions from *Trichilia emetica* and *Opilia amentacea*). Drugs are administered via oral route. Treatment continued for seven days [42].

*(ii) Acute Study (Single Day Study).* Blood samples were collected from rat caudal vein and serum glucose levels were estimated at 0, 1, 3, and 5 h after the extract administration.

*(iii) Subacute Stud (Seven Day Study).* Blood samples were collected from rat caudal vein and serum glucose levels were estimated at 1, 3, 5, and 7 days. Blood glucose levels were determined by god-pod method.

#### 2.10.2. *In Vivo *Hypolipidaemic Potential

*(1) Induction of Experimental Diabetes.* Diabetes was induced by a single intraperitoneal injection of a freshly prepared streptozotocin (STZ) solution (50 mg/kg in citrate buffer 0.01 M, pH 4.5) to overnight-fasted rats [43]. Control rats received normal water alone. Diabetes was identified by polydipsia, polyurea, and measuring nonfasting blood glucose levels 48 h after injection of STZ. Animals which show blood glucose levels more than 250 mg/dL were considered as diabetic rats and used as the experimental animals. 

*(2) Experimental Design.* Adult, male rats of Wistar strain weighing 160–180 g were chosen as animal for present study. They were housed individually in clean, sterile, polypropylene cages under standard conditions and water ad libitum. The animals were acclimatized to the laboratory for one week prior to the start of experiments. The rats were divided into 7 groups comprising of 6 animals in each group as follows: group I: normal rats (controls); group II: diabetic untreated rats; group III: diabetic + glibenclamide treated rats; group IV: normal + *Trichilia emetica* and *Opilia amentacea* treated rats (100 mg/kg of flavonoid-rich fractions from leaves of *Trichilia emetica* and *Opilia amentacea*/day); group V: normal + *Trichilia emetica* and *Opilia amentacea* treated rats (500 mg/kg of flavonoid-rich fractions from leaves of *Trichilia emetica* and *Opilia amentacea*/day); group VI: diabetic + *Trichilia emetica* and *Opilia amentacea* treated rats (100 mg/kg of flavonoid-rich fractions from leaves of *Trichilia emetica* and *Opilia amentacea*/day); and group VII: diabetic + *Trichilia emetica* and *Opilia amentacea* treated rats (500 mg/kg of flavonoid-rich fractions from leaves of *Trichilia emetica* and *Opilia amentacea*/day). Drugs are administered via oral route; treatment continued for 28 days.

*(3) Measurement of Serum Biochemical Parameters.* Blood samples were collected by cardiac puncture. The blood samples without anticoagulant were centrifuged at 3000 rpm for 5 min to obtain plasma or serum. Estimation of serum cholesterol was carried out by the method of [44]. Serum triglycerides were estimated by the method of [45] and HDL cholesterol was estimated by the method of [46]. The VLDL cholesterol was calculated using the formula, TG/5 mg/dL. The serum LDL cholesterol was estimated by the method of [47].

*(4) Estimation of Triglycerides (TG).* Triglycerides in the liver tissue were estimated by modified version method of [48] with slight modifications as given below. Triglycerides were assayed by hydrolyzing them to glycerol and the liberated glycerol was determined. Tissue homogenates were taken and 0.5 mL of 1 N H_2_SO_4_ and 4 mL of chloroform were added. The contents were centrifuged at 1000 rpm for 15 min. The 0.5 of chloroform layer was taken and to 0.4 mL of methanol and 0.1 mL of alkaline barium solutions was added and the contents were heated for 30 min at 80°C; the total volume was made up to 1 mL with 2 N H_2_SO_4_ and centrifuged for 10 min at 1000 rpm. The 0.5 mL of this supernatant was taken and 0.1 mL of sodium periodate was added and shaken well for 1 min; 0.1 mL of sodium arsenate and 5 mL of chromotropic acid reagent were added and heated for 30 min and cooled. The samples were evaluated under wavelength 575 nm spectrophotometrically. The results were finally expressed in mg of triglycerides/gram wet weight of the tissue.

*(5) Estimation of Total Cholesterol.* The total cholesterol content of liver tissue was estimated using Liebermann Burchard reaction as described by [48]. 

*(6) Estimation of Phospholipids.* Phospholipids (PL) in the liver tissue were estimated by the method of [49].

#### 2.10.3. Statistical Analyses

Data were expressed as mean ± standard deviation (SD) of six experiments (*n* = 6). Results were analyzed by one-way ANOVA followed by Dunnett's *t*-test using Prism 4 software. The level of significance was considered at *P* ≤ 0.05.

## 3. Results

### 3.1. Antioxidant Potential

#### 3.1.1. Antioxidant Capacity of *β*-Carotene-Linoleic Acid Assay

**Figure 1:**
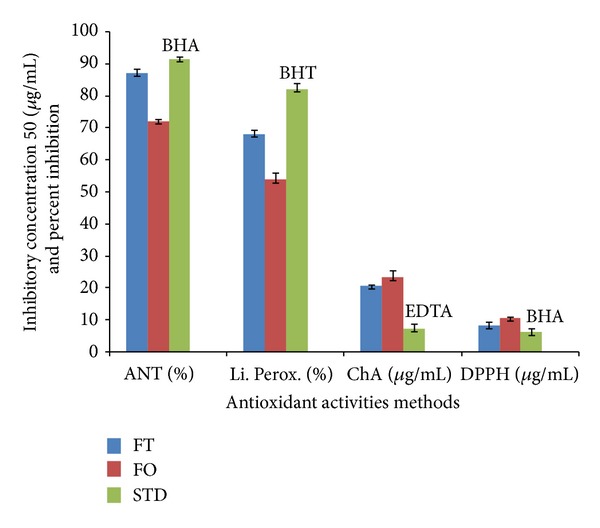
Antioxidant activity in *β*-carotene-linoleate test (ANT), DPPH radical scavenging activity (EC_50_), and metal chelating activity (ChEC_50_) of flavonoid-rich fractions from *Trichilia emetica *(FT) and *Opilia amentacea *(FO) and standards (STD).

The basis of *β*-carotene-linoleic acid assay is degradation of *β*-carotene in reaction with linoleic acid free radical. Antioxidants present in the solution can hinder this reaction and consequently prevent discoloration of *β*-carotene solution. The reduction of absorbance of *β*-carotene-linoleic acid emulsion was shown in presence of the flavonoid-rich fractions. Comparison of the ANT values of the samples (Figure 1) indicates that the flavonoid-rich fractions were less successful at inhibition of bleaching of *β*-carotene emulsion comparatively to BHA (*P* < 0.05 and *P* < 0.001).

#### 3.1.2. Antioxidant of DPPH Radical-Scavenging Activity

The basis of DPPH assay is the discoloration of DPPH^*∙*^ solution in presence of an antioxidant. In its radical form, DPPH absorbs with maximum at 517 nm, but upon reduction with an antioxidant. In this study, flavonoid-rich fractions demonstrated notable antiradical activities albeit lower than the activity of BHA (*P* < 0.001). Results are consigned in Figure 1.

#### 3.1.3. Chelating Activity (ChA)

The chelating ability of the extracts toward ferrous ions was investigated (Figure 1) in presence of ferrozine, Fe^2+^ ion chelator, which upon binding of the metal ion absorbs with maximum at 562 nm. The investigated flavonoid-rich fractions demonstrated significant chelating ability in the present research, although has been found lower than the ability of EDTA (*P* < 0.0001).

#### 3.1.4. Lipid Peroxidation

The endogenous basal of malondialdehyde in the rat liver homogenate was 40.02 mmol/g tissue. After 30 min of incubation FeCl_2_-H_2_O_2_, the incubation of malondialdehyde increase was measured at 535 nm. The inhibition of lipid peroxidation activity by the flavonoid-rich fractions is presented in Figure 1. We noticed that flavonoid-rich fractions showed different statistically significant percentages of inhibition as compared to BHT (*P* < 0.001 and *P* < 0.0001).

### 3.2. *In Vitro* Antihyperglycemia

**Figure 2:**
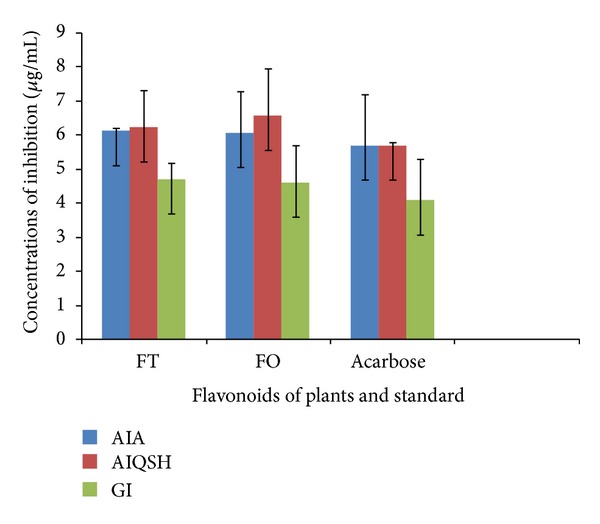
Flavonoid-rich fractions from *Trichilia emetica* (FT) and *Opilia amentacea* (OP) and acarbose (standard) on inhibition of key enzymes (AIA = amylase inhibition screening assay, AIQSH = amylase inhibition assay by quantitative starch hydrolysis, and GI = glucosidase inhibition assay) in carbohydrate metabolism.

In the amylase assay, the positive control acarbose showed an IC_50_ of 5.70 *μ*g/mL and flavonoid-rich fractions exerted an IC_50_ of 6.12 *μ*g/mL for amylase inhibitory activity comparatively to the amylase inhibition assay by quantitative starch hydrolysis where flavonoid-rich fractions exerted an IC_50_ of 6.23 for glucose inhibition activity (*P* < 0.001). In the investigated flavonoid-rich fractions no statistical significance has been observed (IC_50_ of 4.70 *μ*g/mL and IC_50_ of 4.61 *μ*g/mL) compared to the control, although was lower than IC_50_ of acarbose (4.08 *μ*g/mL) for glucosidase inhibition (*P* > 0.05) (Figure 2).

### 3.3. *In Vitro* Antihypertension

**Figure 3:**
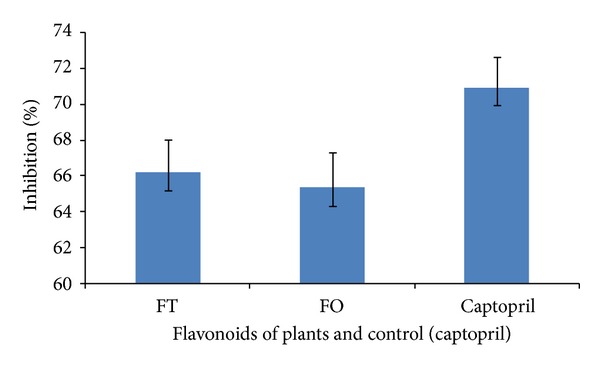
Percent antihypertensive activity upon treatment with flavonoid-rich fractions from *Trichilia emetica* (FT) and *Opilia amentacea* (FO). Captopril was the positive control.

Flavonoid-rich fractions which were tested for antihypertension assay showed >65% inhibition of ACE compared to the control Figure 3. Captopril, which was used as a positive control, gave >70%. Comparison of the flavonoid-rich fractions from *Trichilia emetica *and *Opilia amentacea* values to the control, (Figure 3) showed statistically significant percentages of inhibition in both variants (*P* < 0.05).

### 3.4. Acute Toxicity Study in Mice

It was confirmed the efect of intraperitoneal treatment of aqueous acetone extracts from *Trichilia emetica *and *Opilia amentacea* on mortality, LD_50_ values. The value of LD_50_ is 568.5 mg/kg body weight for intraperitoneal administration for *Trichilia emetica *and 636.2 mg/kg body weight. During 14-day period of acute toxicity evaluation, some signs of toxicity have been observed but were quickly reversible.

### 3.5. *In Vivo* Antihyperglycemia Profile of Flavonoid-Rich Fractions

The results of *in vivo* antihyperglycemia of flavonoid-rich fraction are presented in Tables 1 and 2. We noticed that *Trichilia emetica* (300 mg/kg) possess higher antihyperglycimia parameters results than *Opilia amentacea* and comparatively to the control (*P* < 0.05, *P* < 0.001, and *P* < 0.0001).

### 3.6. *In Vivo* Antihyperlipidaemia Potential of Flavonoid-Rich Fractions

**Figure 4:**
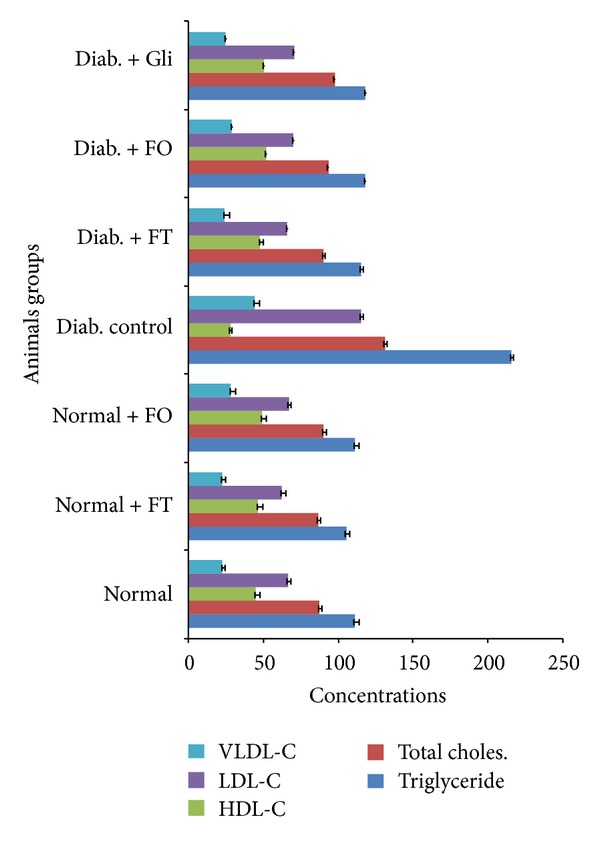
Effect of flavonoids from *Trichilia emetica* (FT) and *Opilia amentacea *(FO) on the concentrations of triglycerides, total cholesterol, HDL-C, LDL-C, and VLDL-C in serum of normal and experimental rats.

**Figure 5:**
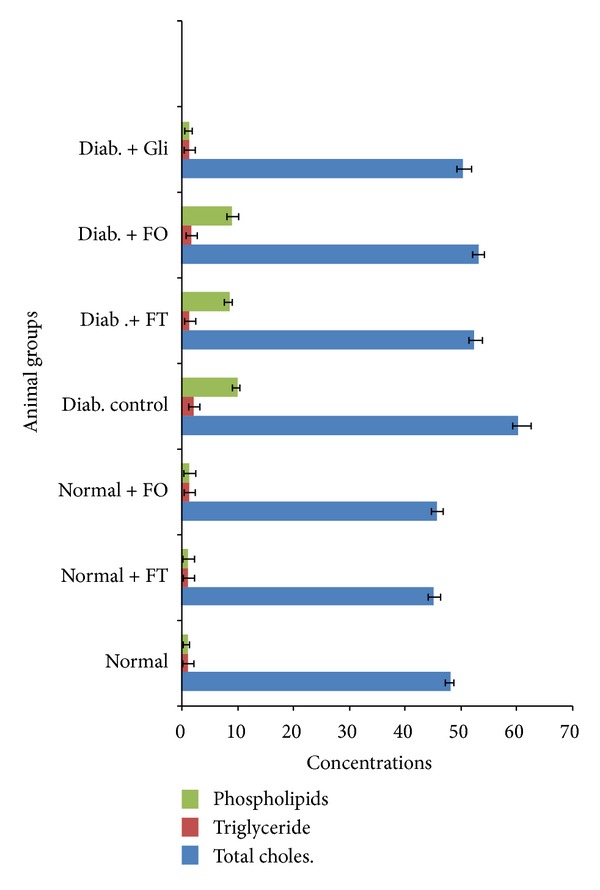
Effect of flavonoid-rich fractions from *Trichilia emetica* (FT) and *Opilia amentacea *(FO) on the concentrations of total cholesterol, triglycerides and phospholipids in the liver of normal and experimental rats.

In this section, we noticed a significant decrease in serum HDL cholesterol levels and a significant elevation in the total cholesterol, triglycerides, and LDL-cholesterol levels in diabetic rats compared to normal rats. Oral administrations of flavonoid-rich fractions for 28 days brought back the levels of serum lipids to near normal levels in diabetic rats and they were restored after administration of flavonoids for a period of 28 days. The total cholesterol, triglycerides, and phospholipids of normal rat hepatic tissue were 48.12 ± 0.53, 1.12 ± 0.10, and 1.21 ± 0.21, respectively, whereas in the diabetic rats these levels are raised to 60.17 ± 2.31, 2.17 ± 0.32, and 10.01 ± 0.33, respectively. The changes of these investigated parameters in the plants after treatment with bioactive fractions were shown in the hepatic tissue: 52.35 ± 1.14, 1.43 ± 0.42, and 8.55 ± 1.1 for *Trichilia emetica* and 53.01 ± 1.10, 1.71 ± 1.10, and 9.02 ± 0.1 for *Opilia amentacea*, respectively (*P* < 0.05). The total cholesterol, triglycerides, LDL-C, and VLDL-C levels of normal rat serum were 87.61 ± 2.01, 111.1 ± 2.51, 66.52 ± 0.51, and 23.2 ± 1.1, whereas in diabetic rats these levels have been raised with the plant bioactive fraction treatment with *Trichilia emetic *extract to 90.27 ± 1.60, 115.28 ± 1.64, 48.21 ± 2.23, and 24.5 ± 1.10 and 118.22 ± 2.2, 93.41 ± 1.81, 70.4 ± 1.12, and 29.43 ± 1.8 for *Opilia amentacea* extract, respectively. The HDL-C levels of normal rats were 45.18 ± 2.26. The levels were regained with the flavonoid-rich fraction treatment to 50.61 ± 1.36. We noticed that extract of flavonoid-rich fractions from *Trichilia emetica* presented better effects on the stabilization of concentrations of total cholesterol, triglycerides, and phospholipids in the liver of experimental rats compared to extract of *Opilia amentacea *(Figures 4 and 5).

## 4. Discussion

Type 2 diabetes is a global health challenge and the WHO has recommended research and use of complementary medicines for the management of this disease. Type 2 diabetes was previously considered as maturity-onset diabetes but, due to increasing rates of obesity, there is an increasing risk of developing this disease in childhood [46, 47].

In this study, flavonoid-rich fractions antioxidant activity was investigated using four assays which cover different aspects of antioxidant activity. Being a relatively stable free radical, DPPH^*∙*^ is frequently used to determine radical-scavenging activity of natural compounds. DPPH assay estimates the ability of sample to scavenge free radicals, species capable of causing damage to natural macromolecules, such as nucleic acids, polysaccharides, and lipids [50]. We noticed that the antiradical activity of flavonoid-rich fractions from *Trichilia emetica* was more pronounced than the activity of the same fractions from *Opilia amentacea*.

In *β*-carotene-linoleic acid assay, the degradation of *β*-carotene occurs in reaction with linoleic acid free radical formed at elevated temperatures. Subsequent loss of conjugation leads to a decrease in absorbance at 470 nm. Antioxidants present in the solution can prevent the degradation of *β*-carotene by reacting with the linoleic acid free radical or any other radical formed in the solution [29]. Thus, in this assay, the capacity of antioxidants to prevent degradation of natural lipids, such as linoleic acid, is measured. The reducing power of a compound, on the other hand, is related to its electron transfer ability and may serve as a significant indicator of its potential antioxidant activity. It estimates the ability of a substance, or an extract which contains it, to donate a proton and thus cause the transformation of free radicals into a less reactive species. In this study, also *Trichilia emetica* was a more successful inhibitor of *β*-carotene bleaching than *Opilia amentacea*. Among the biologically relevant ROS (H_2_O_2_, O_2_^∙−^, and ^*∙*^OH), hydroxyl radicals are the most reactive and dangerous species [51]. Free ferrous iron is sensitive to oxygen and gives rise to ferric iron and superoxide, thereby generating hydrogen peroxide. Thus formed hydrogen peroxide reacts with ferrous iron and generates the hydroxyl radical, which may subsequently oxidize surrounding biomolecules. The investigated flavonoid-rich fractions from *Opilia amentacea* constituents were incapable of chelating ferrous ions in this assay comparatively to the control. *Trichilia emetica* flavonoid-rich fractions, on the other hand demonstrated significant chelating ability. The differences in chemical compositions have probably caused such difference of ferrous ions. The activity of *Trichilia emetica *extracts could certainly be explained by phenolic compounds [27]. Some studies demonstrated that the extracts having higher phenol content also have higher DPPH radical-scavenging activity and other types of antioxidant activities [52–55]. We noticed that antioxidant activity of *Trichilia emetica* was more pronounced in most of the performed assays which leads to conclusion that besides polyphenols some other compounds may at least partly be responsible for the antioxidant activity of investigated extracts.

The inhibition of key enzyme linked to type 2 diabetes, such as *α*-amylase and *α*-glucosidase, has been considered to be an effective strategy to control blood glucose [56]. Agents based on natural products are particularly attractive as side effects are minimal and the therapies are well tolerated compared to the other oral hypoglycemic agents currently available [35, 57]. We noticed that extract with flavonoid-rich fractions from *Trichilia emetica* possessed marked antiamylase activity which may be due to the presence of certain secondary metabolites. In effect, flavonoids, alkaloids, and triterpenoids may be related to the antidiabetic activity of plants. In particular, flavonoids are responsible for variety of pharmacological activities [55]. For example, epicatechin is known to possess insulin-like properties, while epigallocatechin gallate is considered a promising hypoglycemic agent [58]. As shown in previous studies, the enzyme inhibition activity may be related to the polyphenolic content of the plant extract; however, further studies are needed to confirm this.

About antihypertensive property, it is well known that, in recent years, the treatment of hypertension has achieved a breakthrough with the identification of ACE inhibitors as a modern therapeutic tool and considerable interest has focused on the action of ACE inhibitors. ACE inhibitors have potentially improved endothelial function; they are known to increase the plasma concentration of bradykinin, an endothelium-dependent vasodilator, by inhibiting degradation of the peptide [9]. Procyanidins and flavonoids are the major natural products isolated from ethnopharmacologically important plants against *in vitro* ACE inhibitory activity [59, 60]. In addition, other active compounds isolated from medicinal plants include phenylpropanes, xanthones, fatty acids, terpenoids, alkaloids, and peptide amino acids [61]. In the present study, flavonoid-rich fractions from *Trichilia emetica* have shown the best antihypertensive potential compared to the control. This may be connected with capacities of some secondary metabolites like flavonoids.

Nowadays are antihypertensive and antidiabetic effects of these plants known which makes popular a traditional medicine in the developing countries. Medicinal plants are often believed to be harmless because they are natural and commonly used for self-medication without supervision. This increase in popularity and the scarcity of scientific studies on their safety and efficacy have raised concerns regarding toxicity and adverse effects of these remedies [62]. These products of plants contain bioactive principles with the potential to cause adverse effects [63]. The results of the present study indicated that the aqueous acetone extracts of *Trichilia emetica* and *Opilia amentacea* are not toxic. During 14-day period of acute toxicity evaluation some signs of toxicity have been observed, but they were all quickly reversible. According to [64, 65], pharmacological substances where whole LD_50_ less than 5 mg/kg body weight are classified in the range of highly toxic substances, those with LD_50_ between 5 mg/kg body weight and 5000 mg/kg body weight are classified in the range of moderately toxic substances, and those with lethal dose more than 5000 mg/kg body weight not toxic. In this fact, if we refer to this classification we would say that the extracts of *Trichilia emetica* and *Opilia amentacea *are moderately toxic and would be regarded as being safe or have low toxicity.

Traditional plant remedies have been used for centuries in the treatment of diabetes mellitus [66], but only a few have been scientifically evaluated. Alloxan is known for its selective pancreatic islet *β* cell cytotoxicity and has been extensively used to induce diabetes mellitus in animals [67]. Generalized increase in the level of blood glucose during diabetes has been consistently reported both in animal models [68] and humans especially those suffering from insulin dependent diabetes mellitus [69]. In this study, increase in blood glucose level was observed on induction of diabetes mellitus in the rats models, which has been reduced in a dose dependent manner with the highest percentage reduction at 300 mg/kg (Tables 1 and 2). At 3rd hours of exposure in variant with dose of 100 mg/kg did not found any significant antidiabetic activity. The flavonoid-rich fractions at the dose of 500 mg/kg show very significant antidiabetic activity from the first day to seventh day; the dose of 100 mg/kg shows significant antidiabetic activity from the 5th day to 7th day. Our study showed that it is possible that extracts may act by undetermined ways apart from stimulating insulin production from the pancreatic islets since these would have been severely damaged by alloxan. The mechanism of the hypoglycaemic effects of flavonoid-rich fractions remains speculative; therefore further studies are required to unravel the pathway of its hypoglycaemic action and to shed more light on the hypoglycaemic constituents of the plants. It is, however, evident that flavonoid-rich fractions contain hypoglycaemic agents capable of lowering blood glucose level in the alloxan diabetic rats (Tables 1 and 2).

It is well known that the prevalence of hyperlipidaemia among diabetics is increasing worldwide. Alteration in serum lipids profile is known in diabetes, which are likely to increase the risk of coronary heart disease [70]. Lipid profile which is altered in serum of diabetic patients [71] appeared to be a significant factor in the development of premature atherosclerosis through increase in serum triglyceride and total cholesterol levels. The significant reduction in serum cholesterol and total lipids in a dose dependent manner as observed in this experimental work has been confirmed with results of previous reports [72]. The marked hyperlipidaemia that characterizes the diabetic state may be regarded as a consequence of the uninhibited actions of lipolytic hormones on the fat depots [73]. A reduction in lipid profile could be beneficial in preventing diabetic complications as well as improving lipid metabolism in diabetics [74].

Considering flavonoid-rich fractions effects on lipid components [75], it can be assumed a potential hypolipidaemic agent which will be a great advantage both in diabetic conditions as well as in the associated hyperlipidaemic conditions. In effect, the use of synthetic drugs for management of diabetes mellitus has certain adverse effects and therefore there is a need to develop safer and more effective antidiabetic drugs. Consequently, treatment with drugs isolated from plants has an effect on shielding *β* cells and leveling the oscillation in glucose levels [76]. Elevated serum or tissue lipids and lipoproteins are characteristics of uncontrolled diabetes. Type 2 diabetes mellitus is commonly associated with dyslipidemia which is a significant risk factor for the development of cardiovascular diseases [77]. This is in support with results of our present study. In the present, a marked increase in the lipid content of serum and liver was found in STZ induced diabetic rats which is mainly due to the increased mobilization of free fatty acids (FFAs) from peripheral depots [78]. Interestingly, most of the studies with different plant extracts in diabetic rats were supportive of our results [79]. The rise in serum triacylglycerols, cholesterol, and LDL-cholesterol levels in the present study indicates derangement of lipid metabolism and amplified incidence of cardiac dysfunction in diabetic rats. Rise in serum lipids indicates either the defective overproduction or removal (or both) of one or more lipoproteins [80]. An oral administration of flavonoid-rich fractions for a period of 28 days restored the altered levels of lipids (triglycerides, total cholesterol, and phospholipids) in liver tissue as well as in serum. The decreased levels, that is, restoration levels, of cholesterols and triglycerides are due to the presence of glycosides in the flavonoid-rich fractions. The elevated concentrations of cholesterol can enhance the risk of oxidative disease process due to susceptibility of cholesterol to oxidation while it is in circulation. Insulin deficiency or insulin resistance may be responsible for dyslipidemia, because insulin has an inhibitory action on HMG-coA reductase, a key rate-limiting enzyme which is responsible for the LDL particle metabolism with cholesterol-rich content [81]. A shortage of insulin is associated with rise in cholesterol levels due to the increased mobilization of lipids from the adipose tissue to the plasma. The enlarged concentration of FFAs in liver may be due to lipid catalysis which leads to enhanced generation of NADPH and activation of NADPH dependent microsomal lipid peroxidation [82]. In addition, phospholipids are vital components of biomembranes and play an essential role in the triglycerides transport [83]. In diabetic rats, the gigantic levels of PLs may be due to the high levels of FFAs and total cholesterol [84].

## 5. General Remarks and Conclusion

The present study assesses the antidiabetic potential of flavonoid-rich fractions from *Trichilia emetica* and *Opilia amentacea*. These plants can be promising agents with good antioxidant activity for the management of hyperglycemia and can be used also in the antihypertensive therapy. However, these results require further investigation to better understand biochemical nature of these effects as they may provide leads for the discovery of new drugs for the management of diabetes type 2 with minimal side effects. Further studies are in progress with isolating these active compounds which are responsible for the antidiabetic and antihypertensive properties.

## Figures and Tables

**Table 1:** Effect of flavonoid-rich fractions of *Trichilia emetica* (FT) and *Opilia amentacea* (FO) on serum glucose level for acute study (single day study).

	0 h	1 h	3 h	5 h
Normal control	84.12 ± 1.31	85.2 ± 2.18	85.30 ± 1.10	85.10 ± 1.99
Diabetic control	177.1 ± 2.23	179.3 ± 1.30	183.5 ± 2.40	186.1 ± 2.10
Standard	174.1 ± 1.20	165.1 ± 2.21*	152.1 ± 1.40***	144.3 ± 3.20***
FT (100 mg/kg)	176.4 ± 3.08	174.2 ± 2.12	166.4 ± 2.63**	164.3 ± 2.39**
FT (300 mg/kg)	172.5 ± 1.50	177.2 ± 1.30	153.5 ± 1.54***	144.2 ± 2.40***
FO (100 mg/kg)	176.9 ± 1.1	178.1 ± 2.10	166.7 ± 1.33**	167.1 ± 2.37**
FO (300 mg/kg)	173.1 ± 0.10	180.2 ± 3.20	155.2 ± 1.24***	146.6 ± 2.71***

Values are mean ± SEM; *N* = 6. *P < 0.05, **P < 0.001, and ***P < 0.0001 versus diabetic control.

**Table 2:** Effect of flavonoid-rich fractions of *Trichilia emetica* (FT) and *Opilia amentacea *(FO) on blood glucose level for subacute study (multiday study).

	0 day	1 day	3 day	5 day	7 day
Normal control	84.60 ± 1.10	85.68 ± 1.00	84.33 ± 2.10	84.57 ± 1.00	84.53 ± 1.52
Diabetic control	176.2 ± 3.30	183.2 ± 2.31	192.4 ± 2.10	198.2 ± 2.10	203.2 ± 2.04
Standard	174.1 ± 2.20	154.2 ± 1.37**	145.6 ± 2.03***	128.2 ± 2.53***	107.1 ± 1.10***
FT (100 mg/kg)	176.1 ± 1.20	170.1 ± 2.02*	164.2 ± 1.22**	160.4 ± 1.00**	156.6 ± 2.67**
FT (300 mg/kg)	172.5 ± 2.57	155.2 ± 1.42**	145.3 ± 1.10***	128.4 ± 2.17***	113.1 ± 3.53***
FO (100 mg/kg)	176.8 ± 3.10	174.3 ± 1.12*	167.5 ± 1.20**	163.0 ± 1.01**	158.2 ± 2.60**
FO (300 mg/kg)	173.1 ± 1.53	157.1 ± 1.10**	146.9 ± 2.23***	128.6 ± 1.10***	118.7 ± 1.51***

Values are mean ± SEM; *N* = 6. *P < 0.05, **P < 0.001, and ***P < 0.0001 versus diabetic control.
